# Association of bone mineralization markers with dietary nutrient intake in adolescents with and without biochemical osteomalacia

**DOI:** 10.3389/fnut.2023.1206711

**Published:** 2023-07-17

**Authors:** Nasser M. Al-Daghri, Shaun Sabico, Kaiser Wani, Syed Danish Hussain, Sobhy Yakout, Naji Aljohani, Suma Uday, Wolfgang Högler

**Affiliations:** ^1^Chair for Biomarkers of Chronic Diseases, Biochemistry Department, College of Science, King Saud University, Riyadh, Saudi Arabia; ^2^Obesity Endocrine and Metabolism Center, King Fahad Medical City, Riyadh, Saudi Arabia; ^3^College of Medicine, Alfaisal University, Riyadh, Saudi Arabia; ^4^Department of Endocrinology and Diabetes, Birmingham Women’s and Children’s Hospital, Birmingham, AL, United Kingdom; ^5^Institute of Metabolism and Systems Research, University of Birmingham, Birmingham, AL, United Kingdom; ^6^Department of Pediatrics and Adolescent Medicine, Johannes Kepler University Linz, Linz, Austria

**Keywords:** biochemical osteomalacia, bone mineralization, vitamin D, calcium, phosphorous, alkaline phosphatase, dietary recall

## Abstract

**Background:**

Dietary intake is widely known to play a crucial role in achieving peak bone mass among children and adolescents. Unfortunately, this information is lacking among Arab adolescents, an understudied demographic that has recently been observed to have a high prevalence of abnormal mineralization markers [low serum 25(OH)D, high serum alkaline phosphatase (ALP), low calcium (Ca) and/or inorganic phosphate (Pi)] suggestive of biochemical osteomalacia (OM, defined as any 2 of the 4 parameters). In order to fill this gap, we aimed to evaluate the associations of serum markers of biochemical OM with dietary intake of macronutrients, vitamins and trace minerals.

**Methods:**

Saudi adolescents (*N* = 2,938, 57.8% girls), aged 12–17 years from 60 different schools in Riyadh, Saudi Arabia were included. Dietary intake of nutrients was calculated following a semi-quantitative 24 h dietary recall over 3 weekdays and 1 weekend-day using a validated food frequency questionnaire. Compliance to reference daily intake (RDI) of macronutrients, vitamins and trace minerals were calculated. Fasting blood samples were collected and circulating levels of 25(OH)D, ALP, Ca, and Pi were analyzed.

**Results:**

A total of 1819 (1,083 girls and 736 boys) adolescents provided the dietary recall data. Biochemical OM was identified in 175 (9.6%) participants (13.5% in girls, 3.9% in boys, *p* < 0.01) while the rest served as controls (*N* = 1,644). All participants had serum 25(OH)D levels <50 nmoL/L. Most participants had very low dietary intakes of Ca (median ~ 290 mg) and vitamin D (median ~ 4 μg) which are far below the RDI of 1,300 mg/day and 20 μg/day, respectively. In contrast, excess dietary intakes of Pi, Na, K, and Fe were observed in all participants. In the biochemical OM group, thiamine and protein intake were significant predictors of serum 25(OH)D, explaining 4.3% of the variance perceived (*r* = 0.23, adjusted *r*^2^ = 4.3%, *p* = 0.01). Among controls, dietary vitamin C and vitamin D explained 0.6% of the total variation in serum 25(OH)D (*r* = 0.09, adjusted *r*^2^ = 0.6%, *p* = 0.004).

**Conclusion:**

Arab adolescents do not meet the RDI for dietary Ca and vitamin D, and none have sufficient vitamin D status (25(OH)D levels >50 nmol/L) but they exceed the RDI for dietary Pi. Interpreting these data in the light of the increased prevalence of rickets in Arab countries, food fortification to optimise vitamin D and Ca intake in Saudi adolescents should be considered.

## 1. Introduction

Sustaining a healthy diet has been consistently considered a major factor for preventing major chronic non-communicable diseases (diabetes, hypertension, atherosclerosis, etc.) and mortality (1, 2). Despite this established fact, the most recent Global Nutrition Report (2022) has implicated that among the greatest global public health challenges in modern times are poor or unhealthy diet characterized by over consumption of highly processed foods that are low in nutrients, and malnutrition (3). In fact, a recent large-scale data gathered from 185 countries between 1990 and 2018 demonstrated that while diet quality was considered acceptable for infants and children globally, this pattern appears to worsen over time among older children and adolescents, having relatively lower quality scores compared to adults. This poor-quality score among adolescents needs to be addressed as it can substantially affect their bone health, among others, as it has been established that peak bone mass can be significantly altered by nutrition aside from genetics (4). Dietary calcium and protein in particular impact bone capital, both of which are found in dairy products, and children who consume fewer dairy products are more susceptible to fragility fracture as adults (5). Unfortunately, this poor dietary pattern is most notable in the Middle East and North Africa (MENA) with folate, iron and vitamin D being the most common micronutrient deficiencies (6, 7). Vitamin D deficiency is overwhelmingly common in this region, particularly in Saudi Arabia (SA) where the prevalence remains high, despite abundant year-round sunshine (8). Common factors for vitamin deficiency in SA include extreme heat during summer season, as well as cultural/religious factors such as the full covering of (mostly) women when going outdoors (8). In SA alone, vitamin D deficiency has been linked to a host of extra-skeletal disorders such as cardiovascular disease (9), type 2 diabetes mellitus (10), and premature biological ageing (11). Most of these studies however were conducted in adults, and available data on extra-skeletal effects of vitamin D deficiency in younger Arab populations are scarce.

Among the more well-known effects of chronic, severe vitamin D deficiency in children and adolescents are nutritional rickets and osteomalacia (OM) (12, 13). Their exact prevalence is difficult to ascertain, as they mostly remain clinically underdiagnosed in apparently healthy children and adolescents until symptoms such as hypocalcaemic complications, bone pain, stunted growth and skeletal deformities become apparent (14). The gold standard to confirm a diagnosis of OM is taking a transiliac bone biopsy, which due to its invasive nature is rarely performed. Therefore, non-invasive alternatives such as biochemical markers of OM are gaining more interest lately, despite being an old concept (15). Rickets and OM produce an identical, typical biochemical disease signature (above all elevated levels of parathyroid hormone and alkaline phosphatase). Several operational definitions of biochemical OM have been proposed for epidemiologic studies (15–17). Despite the absence of large-scale studies, nutritional rickets and OM have been reported to be increasing globally, especially among high-risk populations such as dark-skinned individuals and migrant populations (18). To allow early recognition of these hypomineralization disorders, it is therefore essential to establish diagnostic biomarkers.

We have previously investigated the prevalence of biochemical OM, defined as having any 2 of the 4 circulating serum markers of impaired mineralization, namely low 25OH vitamin D (25(OH)D < 30 nmoL/L), elevated alkaline phosphatase (ALP) for age and sex, low calcium (Ca, <2.1 mmoL/L) or low inorganic phosphate (Pi, age and sex-configured values) in a cohort of 2,938 Arab adolescents aged 12–17 years. This study found an over-all prevalence of biochemical OM of 10% (15% in girls and 4% in boys) (19).

Given that 1 in every 10 Arab adolescent in SA has biochemical OM, it is crucial to investigate the influence of diet in the bone health of this understudied population, which, to the best of our knowledge, has never been undertaken on a large-scale. In the present study, we evaluated dietary recall data in the same cohort, to determine the differences in dietary intake of macronutrients, vitamins and trace minerals in Arab adolescents with and without biochemical OM and explore the association of these nutrients with OM based on biomarkers of bone mineralization.

## 2. Methods

This observational study is part of a large project aimed at establishing the prevalence of biochemical OM among Arab adolescents, which was conducted between September 2019 and March 2021 (19). The study was done in collaboration with the Ministry of Education in SA. Healthy adolescent boys and girls (no acute medical condition or signs and symptoms that required immediate clinical attention, physically and mentally able to engage in school activities) aged 12–17 years enrolled in more than 60 preparatory and secondary schools in Riyadh, SA, were actively recruited to determine the prevalence of biochemical OM (19), and its association with dietary intake of vitamins and minerals in this population (present study). Institutional Review Board (IRB) approval was obtained from the College of Medicine, King Saud University, KSU, Riyadh, SA (E-21-6,095, approved 18 January 2019; amended 7 April 2022). Only adolescents who provided consent/assent, blood samples and dietary recall information were included. Participants were excluded if they did not consent and/or were medically unfit (with physical and/or mental diseases/disabilities that can interfere with blood and data collection) to participate. Expatriate (non-Saudi) adolescents were excluded by default as they were mostly enrolled in community/private schools not covered by the government.

### 2.1. Data collection

Via flyers and school announcement, interested participants were invited to visit the primary care center nearest to their respective schools in the morning and preferably on a fasted state (8 h minimum). At the primary care center, a trained research nurse, in conjunction with the in-house primary care physician, screened for eligibility and consented, as detailed in previous studies involving school participants (20, 21). Following screening, anthropometric measurements and blood samples were collected. Height (cm) and weight (kg), together with waist (cm) and hip circumferences (cm) were measured using standardized tools with the participants wearing light clothing. Blood pressure (BP, mmHg) was measured twice using appropriate cuffs with a 5 min resting interval and the mean was noted. Body mass index (BMI, kg/m^2^) and waist-hip-ratio (WHR) were calculated. LMS method was used to calculate BMI *z*-scores by using growth reference data for boys and girls for aged 5–19 years (22).

### 2.2. Blood sample collection and analysis

Fasting venous blood samples were collected, centrifuged on-site and labelled for immediate transport with packed ice to the Chair for Biomarkers of Chronic Diseases (CBCD) in KSU, Riyadh, SA, where all sample analysis was conducted. Fasting blood and serum samples upon arrival were immediately stored at −80°C for later use. Markers of bone mineralization which include circulating 25(OH)D, Ca, Pi, alanine transaminase (ALT) and ALP were measured using available facilities in CBCD which are routinely calibrated, as described in detail previously (19). Briefly, serum 25(OH)D was quantified using a chemiluminescent immunoassay (DiaSorin, Saluggia, Italy) [inter-assay coefficient of variation (CV) of 10.6% and intra-assay CV of 5.4%, with a lower detection limit of 10 nmol/L], while trace minerals (Pi, Ca and iron (Fe)), albumin and ALP were assessed using a biochemical analyzer (Konelab, Vintaa, Finland) that was routinely calibrated using manufacturer-provided quality control samples (Thermo Fisher Scientific, Espoo, Finland). The serum Ca*P product was calculated (in mmol^2^).

### 2.3. Dietary information

Participants were given the option to provide dietary information on the same day or within the week following blood extraction. A semi-quantitative validated 24 h food recall (23) was administered in a structured face-to-face interview with a trained dietician. Detailed information of all foods and beverages consumed by the participant in a 24 h period over 3 weekdays and one weekend-day was gathered as done in previous studies (24). For accuracy of data collection, participants were shown food model illustrations with varying dish, cup and utensil sizes to capture actual intake. The dietary recall collected was then entered digitally on a food software (ESHA’s Food Processor^®^ Nutrition Analysis, OR, United States) to assess participant’s total energy (kcal), macronutrients (fat, protein, carbohydrates (CHO) and fiber), trace minerals (Na, K, Ca, Pi, and Fe) and vitamin intakes (vitamin A, thiamine, riboflavin, vitamins B12, C and D) relevant to the present study. Nutrient recommendations were based on those published by the US Food and Nutrition Board (FNB) of the National Academy of Medicine (25). Reference daily intakes (RDI) were plotted with the obtained nutrient intake of participants to compare whether their consumption was within recommendations. All results obtained were shared individually with the participant and/or parent/guardian, together with the blood results and accompanying advice from the primary care physician. Adequacy index was calculated as daily nutrient intake/recommended allowance for sex and age × 100 and compared with the RDI of FNB (25, 26).

### 2.4. Biochemical OM

Participants were stratified according to the definition of biochemical OM, which, in the present study, used the previous operational classification of any two of the four serum markers of impaired mineralization (19) which include low 25(OH)D (<30 nmol/L), elevated ALP according to age- and sex-specific reference ranges (27), and either low Ca (<2.1 mmol/L) and/or low Pi according to pediatric ranges (28) (Supplementary Table S1).

### 2.5. Data analysis

Data were analyzed using SPSS v21.0 (Chicago, Illinois). Normal continuous variables were presented as mean ± standard deviation (SD) and non-normal continuous variables as median (quartile 1–quartile 3). Frequencies were presented in percentage (%). Independent sample *t*-test and Mann–Whitney U test were used to compare participants with biochemical OM and those without, adjusted for sex. Pearson bivariate correlation analysis was also done to determine the associations between markers of impaired mineralization related to biochemical OM and nutritional intake (energy, macronutrients, vitamins, and trace minerals) in all participants and according to biochemical OM status. Lastly, stepwise regression analysis was applied to identify significant predictors using the markers of biochemical OM (Ca, Pi, 25(OH)D and ALP) as dependent variables and the dietary nutrient intake as independent variables. Value of *p* was considered significant at <0.05.

## 3. Results

**Figure 1:**
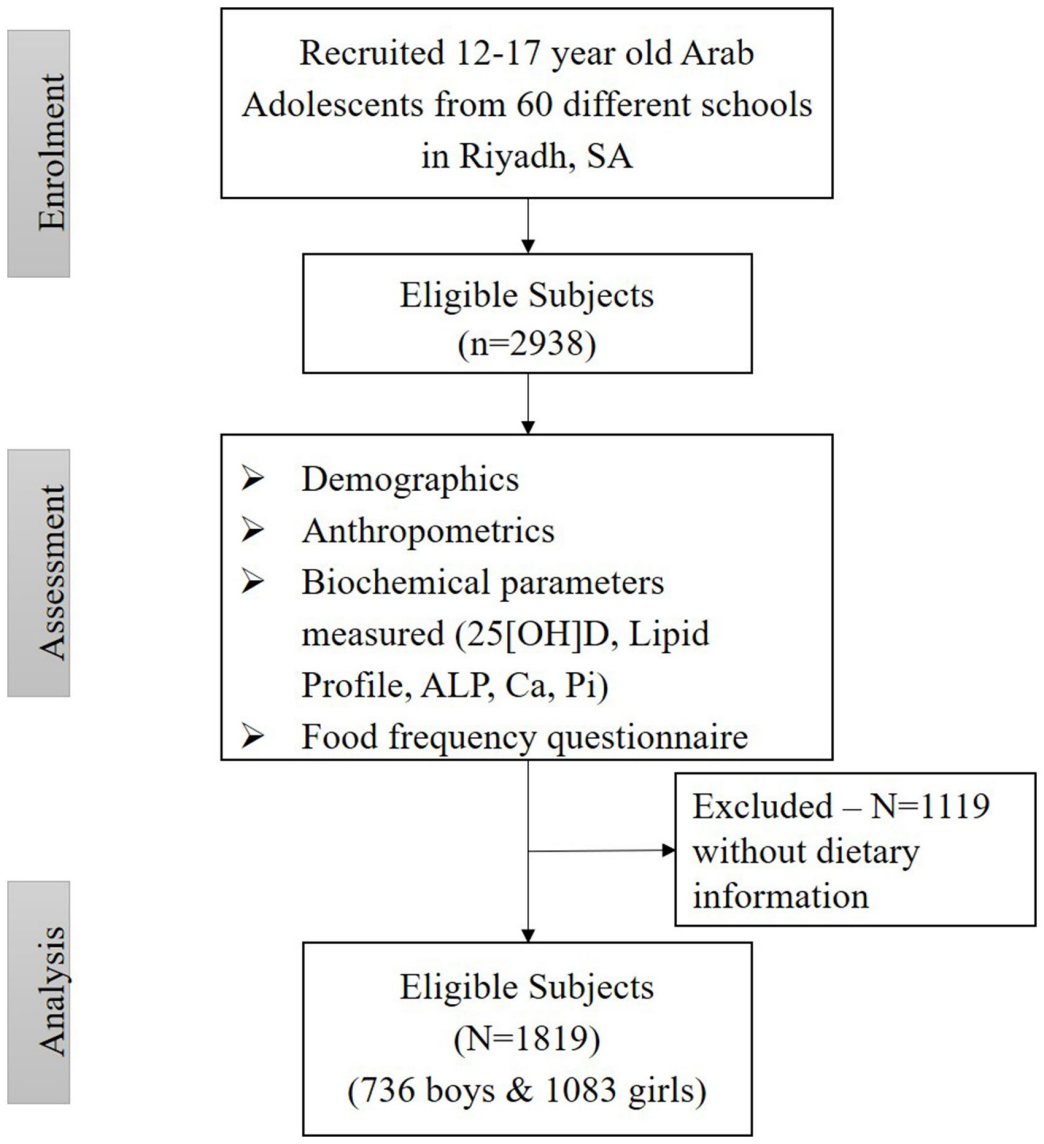
Participant flow chart.

Out of the original 2,938 adolescents who provided blood samples, only 62% or *N* = 1819 completed the 24 h dietary recall questionnaires. Hence, a total of 1819 (1,083 girls and 736 boys) were included in the present observational study (Figure 1), of whom 175 (9.6%) had biochemical OM (13.5% in girls, 3.9% in boys, *p* < 0.01). None of the participants had sufficient 25(OH)D level (above or equal to 50 nmoL/L).

### 3.1. Clinical characteristics according to biochemical OM status

Table 1 shows the differences in anthropometric indices and biochemical profiles of adolescents with biochemical OM (*N* = 175) and without (*N* = 1,644 controls). Girls significantly outnumber the boys in the biochemical OM group (83% versus 17%; *p* < 0.001). There were no significant differences in age and anthropometrics in both groups even after adjusting for sex. Systolic BP was noted to be higher in the control group but lost significance after adjustments for sex. Unadjusted comparisons in markers of biochemical OM showed significantly higher circulating levels of Ca, Pi, Ca*P, ALP, 25(OH)D, ALT, and Fe in the control group as compared to the biochemical OM group (Value of *p*s < 0.05). However, after adjusting for sex, only serum Ca, Pi, Ca*P product and 25(OH)D remained significantly higher in the control group (Value of *p*s < 0.001). As for % adequacy, lower intakes were observed only in dietary vitamin D and Ca among the dietary vitamins and minerals assessed (Table 2).

**Table 1:** Anthropometric and biochemical characteristics of participants.

Parameters	Control	Biochemical OM	Value of *p*	Adjusted *p*-value
*N*	1,644	175		
Age (years)	15.0 ± 1.7	14.8 ± 1.7	0.08	0.28
Boys	707 (43.0)	29 (16.6)	<0.001	–
Girls	937 (57.0)	146 (83.4)		
Height (cm)	159.6 ± 10.3	158.5 ± 9.1	0.20	0.80
Weight (kg)	61.2 ± 16.5	62.2 ± 15.9	0.44	0.10
BMI (kg/m^2^)	23.9 ± 5.7	24.6 ± 5.9	0.10	0.09
BMI *Z*-score	0.0 ± 1.0	0.0 ± 0.1	0.51	0.61
Underweight	93 (5.7)	8 (4.6)	0.24	0.58
Overweight	464 (28.2)	53 (30.3)	0.38	0.85
Waist (cm)	73.2 ± 16.3	72.6 ± 15.1	0.69	0.26
Waist *Z*-score	0.54 ± 1.19	0.56 ± 1.16	0.78	0.22
Central Obesity	615 (37.4)	66 (37.7)	0.82	0.49
Hips (cm)	86.3 ± 18.0	87.4 ± 18.9	0.46	0.18
WHR	0.9 ± 0.1	0.8 ± 0.1	0.20	0.47
Systolic BP (mmHg)	116.3 ± 16.4	113.2 ± 17.8	0.03	0.21
Diastolic BP (mmHg)	72.4 ± 12.1	72.8 ± 12.1	0.68	0.60
Ca (mmol/L)	2.6 ± 0.3	2.3 ± 0.4	<0.001	<0.001
Pi (mmol/L)	1.5 ± 0.4	1.2 ± 0.3	<0.001	<0.001
Ca*P Product	3.8 ± 1.2	2.7 ± 1.0	<0.001	<0.001
ALP (U/L)	64.2 (43.3–91.5)	63.9 (42.3–98.9)	<0.001	0.11
25(OH)D (nmol/L)	33.3 (23.9–44.7)	21.9 (17.5–25.7)	<0.001	<0.001
ALT (U/L)	11.0 (8.1–15.5)	11.0 (7.3–17.2)	0.02	0.97
Fe (μg/L)	876.8 (607.5–1099.9)	743.5 (476.6–1151.5)	<0.001	0.76

Data presented as mean ± SD for normal variables and Median (Quartile 1–Quartile 3) for non-normal variables; *p* < 0.05 adjusted for sex considered significant.

**Table 2:** Nutrient intake according to biochemical osteomalacia status.

Parameters	Intake	Adequacy (%)	RDI*	Control (*N* = 1,644)	Biochemical OM (*N* = 175)	Adj. *p*-value
	Mean (SE)	Mean (SE)		Median (IQR)	Median (IQR)	
Energy (Kcal)	7290.9 (538.3)	364.5 (26.9)	2000	3212.3 (1754–5,746)	3348.2 (1841–6,757)	0.27
Fat (g)	436.3 (39.1)	559.7 (50.1)	78 g	154.1 (80–304)	175.4 (101–374)	0.36
Protein (g)	345.5 (42.4)	691.6 (85.0)	50 g	113.3 (67–220)	124.6 (66–267)	0.23
CHO (g)	655.1 (22.8)	238.2 (8.3)	275 g	427.3 (215–726)	418.8 (214–834)	0.55
Fiber (g)	45.0 (1.6)	162.8 (5.8)	28 g	29.6 (14–52)	28.6 (15–61)	0.69
Minerals						
Na (mg)	8119.8 (385.8)	541.3 (25.7)	1,500 mg	4087.7 (2347–8,774)	4680.1 (2244–10,981)	0.45
K (mg)	11378.3 (635.8)	242.1 (13.5)	4,700 mg	6471.2 (3220–11,214)	6615.8 (3083–12,781)	0.58
Ca (mg)	612.9 (33.8)	47.5 (2.6)	1,300 mg	293.5 (136–513)	265.0 (103–536)	0.42
Pi (mg)	5106.2 (431.1)	408.5 (34.5)	1,250 mg	2245.9 (1399–3,922)	2256.7 (1426–4,180)	0.52
Fe (mg)	79.7 (6.6)	454.0 (37.7)	18 mg	40.8 (20–78)	43.8 (18–82)	0.59
Vitamins						
Vitamin A (μg)	2030.8 (76.3)	225.6 (8.5)	900 μg	1039.1 (506–2,306)	1319.3 (456–2,610)	0.6
Thiamine (mg)	4.7 (0.4)	440.6 (41.1)	1.2 mg	1.6 (0.9–3.3)	1.7 (0.9–4.6)	0.09
Riboflavin (mg)	13.9 (2.5)	1095.4 (194.3)	1.3 mg	4.1 (2.2–9)	5.0 (2.8–12)	0.15
Vitamin B12 (μg)	79.1 (26.9)	3388.5 (1151.8)	2.4 μg	10.2 (5–21)	9.9 (4.7–26)	0.41
Vitamin C (mg)	206.7 (10.2)	231.9 (11.4)	90 mg	111.6 (44–244)	103.7 (39–202)	0.97
Vitamin D (μg)	8.1 (0.6)	44.0 (3.5)	20 μg	4.0 (2–8)	4.2 (2–10)	0.45

Data presented as median (Q1–Q3); *p*-value obtained from Mann–Whitney U test; *p* < 0.05 adjusted for sex considered significant; *Based on reference caloric intake (2,000 calories) for adults and children aged 4 years and above (21).

### 3.2. Dietary intake according to biochemical OM status

Differences in caloric and nutrient intake in those with and without biochemical OM are shown in Table 2. No differences were observed in macro- and micronutrient intake in both groups even after adjusting for sex. Age-appropriate RDI for each vitamin and mineral were compared to the actual median intake values obtained and showed that for all participants, excess intake of dietary Na, K, Pi, and Fe were observed. In contrast, none of the participants met the RDI for Ca. In terms of vitamin intake, participants were able to meet the RDI for most vitamins (A, thiamine, riboflavin, vitamins B12 and C) with the exception of vitamin D.

### 3.3. Associations of markers of biochemical OM with nutrient intake

Bivariate associations of dietary intake and markers of biochemical OM for all participants are presented in Table 3. Modest but significant inverse associations were observed between serum Ca and dietary fiber (*r* = −0.06; *p* < 0.05) as well as dietary Na, K, Pi, Fe, and vitamin A (all *r* = −0.05; *p* < 0.05). On the other hand, serum Pi was significantly and inversely associated with all macronutrients, as well as most vitamins and minerals with the exception of Ca, riboflavin and vitamin D. Similar inverse and significant associations were also observed between circulating serum ALP and macronutrients as well as most trace minerals with the exception of Ca. Serum ALP was the only marker for biochemical OM associated with dietary vitamin D intake (*r* = −0.06; *p* < 0.01), together with vitamins A, B12, and C. Lastly, serum 25(OH)D was not significantly associated with any of the macronutrients, vitamins and minerals (Table 3).

**Table 3:** Correlations between nutrient intake adequacy (%) and markers of biochemical osteomalacia (all participants).

Nutrient parameters	Ca	Pi	ALP	25(OH)D
Energy (Kcal)	−0.04	−0.07**	−0.11**	0.01
Fat (g)	−0.05*	−0.08**	−0.11**	0.00
Protein (g)	−0.03	−0.07**	−0.11**	0.01
CHO (g)	−0.04	−0.06*	−0.10**	0.02
Fiber (g)	−0.04	−0.05*	0.01	0.01
Minerals				
Na (mg)	−0.05*	−0.05*	−0.12**	0.00
K (mg)	−0.05*	−0.07**	−0.12**	0.01
Ca (mg)	0.00	0.00	−0.01	0.02
P (mg)	−0.05	−0.06**	−0.13**	0.00
Fe (mg)	−0.05*	−0.06**	−0.06*	0.00
Vitamins				
Vitamin A (μg)	−0.05	−0.06*	−0.12**	0.00
Thiamine (mg)	0.03	−0.06*	0.04	−0.05*
Riboflavin (mg)	−0.02	−0.02	−0.01	−0.03
Vitamin B12 (μg)	0.00	−0.02	0.01	−0.03
Vitamin C (mg)	−0.02	−0.05*	−0.05	0.03
Vitamin D (μg)	−0.01	0.01	−0.01	−0.07**

Data presented as correlation coefficient (*R*); ** and * indicates significance at <0.01 and <0.05, respectively.

Stratification according to biochemical OM status revealed significant associations between markers of interest [Ca, Pi, ALP, and 25(OH)D] and dietary intake (macronutrients, trace minerals, and vitamins; Table 4) which were not observed when all participants were considered. In the control group, only dietary fiber was inversely and significantly associated with serum Ca (*r* = −0.05; *p* < 0.05). Significant inverse associations with serum Pi were also observed with all macronutrients as well as dietary K, Pi, Fe, vitamin A, thiamine, and vitamin C. Similar significant but stronger inverse associations were seen with serum ALP and macronutrients, all minerals except Ca and most vitamins except thiamine and riboflavin. Again, no associations were elicited between serum 25(OH)D and dietary intake in the control group. In the biochemical OM group, no significant associations were seen between serum Ca and dietary intake of all nutrients assessed. Serum Pi in the biochemical OM group was not associated with any of the dietary vitamins, but was inversely associated with most minerals except Ca, as well as CHO and energy. Lastly, 25(OH)D showed significant positive associations with all macronutrients only after stratification and in the biochemical OM group. These significant positive associations were also observed in all minerals except Ca as well as all vitamins except thiamine (Table 4).

**Table 4:** Correlation between markers of biochemical OM and nutrient intake nutrient intake adequacy (%) according to OM status.

Nutrient parameters	Controls				Biochemical OM			
	Ca	Pi	ALP	25(OH) D	Ca	Pi	ALP	25(OH)D
Energy (Kcal)	−0.03	−0.06*	−0.11**	−0.01	−0.07	−0.15*	−0.12	0.21**
Fat (g)	−0.03	−0.07**	−0.11**	−0.01	−0.11	−0.11	−0.15	0.20**
Protein (g)	−0.02	−0.06*	−0.11**	−0.01	−0.07	−0.14	−0.13	0.23**
CHO (g)	−0.03	−0.05	−0.10**	0.01	−0.08	−0.15*	−0.14	0.19*
Fiber (g)	−0.04	−0.05	0.01	0.00	−0.02	−0.03	0.03	0.13
Minerals								
Na (mg)	−0.04	−0.04	−0.12**	−0.01	−0.11	−0.17*	−0.14	0.20**
K (mg)	−0.04	−0.06*	−0.12**	0.00	−0.12	−0.16*	−0.16*	0.21**
Ca (mg/day)	0.02	0.00	0.01	0.00	−0.07	−0.04	−0.02	0.13
Pi (mg)	−0.03	−0.06*	−0.13**	−0.02	−0.10	−0.15	−0.15*	0.22**
Fe (mg)	−0.04	−0.06*	−0.06*	−0.01	−0.08	−0.11	−0.02	0.16*
Vitamins								
Vitamin A (μg)	−0.04	−0.05*	−0.12**	−0.02	−0.12	−0.13	−0.13	0.22**
Thiamine (mg)	0.02	−0.03	0.05	−0.04	0.08	−0.16	0.06	−0.05
Riboflavin (mg)	−0.01	0.00	−0.01	−0.04	−0.03	−0.04	0.04	0.13
Vitamin B12 (μg)	0.02	−0.01	0.03	−0.04	−0.02	−0.01	−0.02	0.13
Vitamin C (mg)	−0.03	−0.05*	−0.06*	0.02	0.01	−0.03	−0.02	0.14
Vitamin D (μg)	0.01	0.03	0.01	−0.09**	−0.13	−0.05	−0.05	0.13

Data presented as Pearson correlation coefficient; ** and * indicates significance at <0.01 and <0.05, respectively.

**Figure 2:**
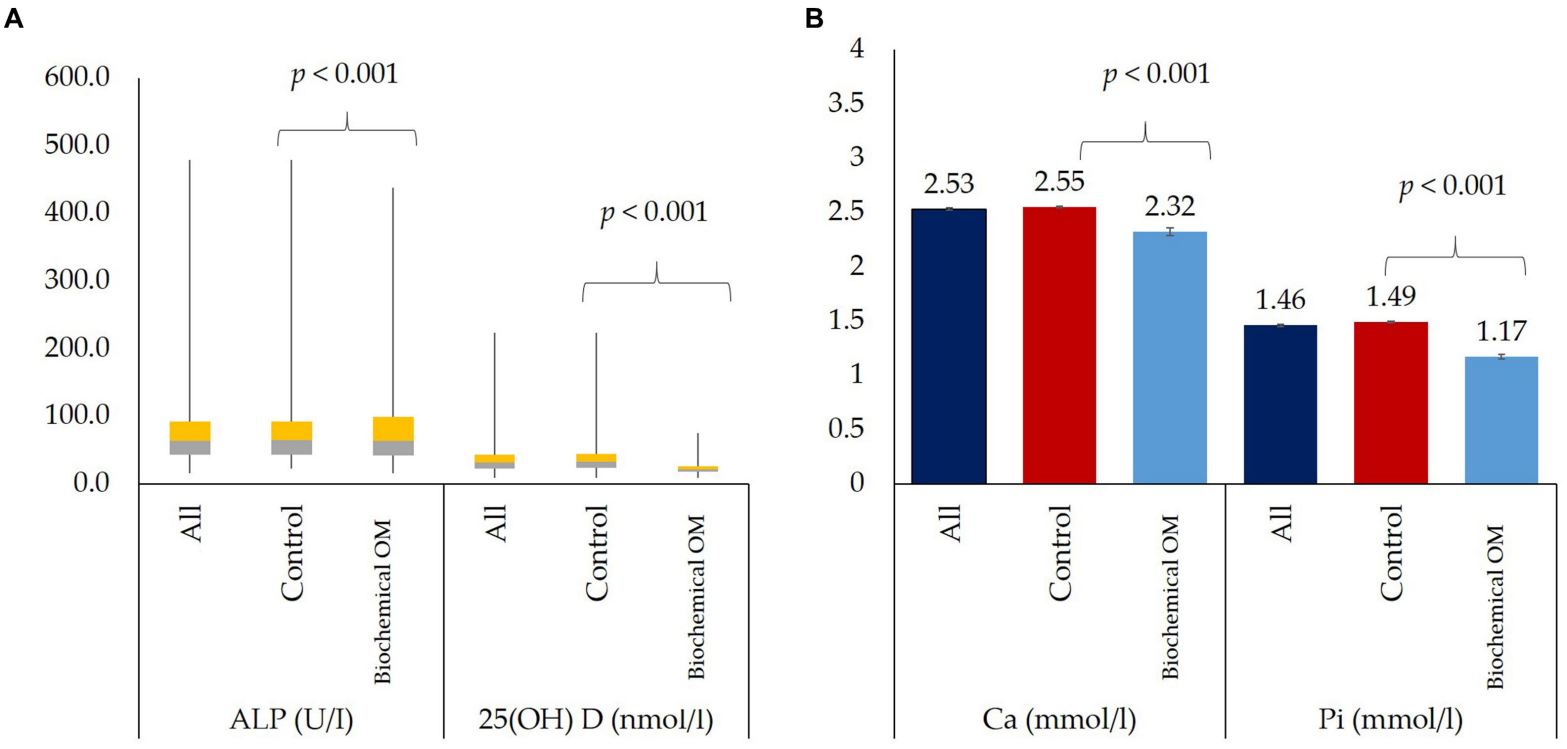
**(A)** Median serum ALP and 25(OH)D of all participants and according to biochemical OM status and **(B)** Mean serum Ca and Pi of all participants and according to biochemical OM status.

**Figure 3:**
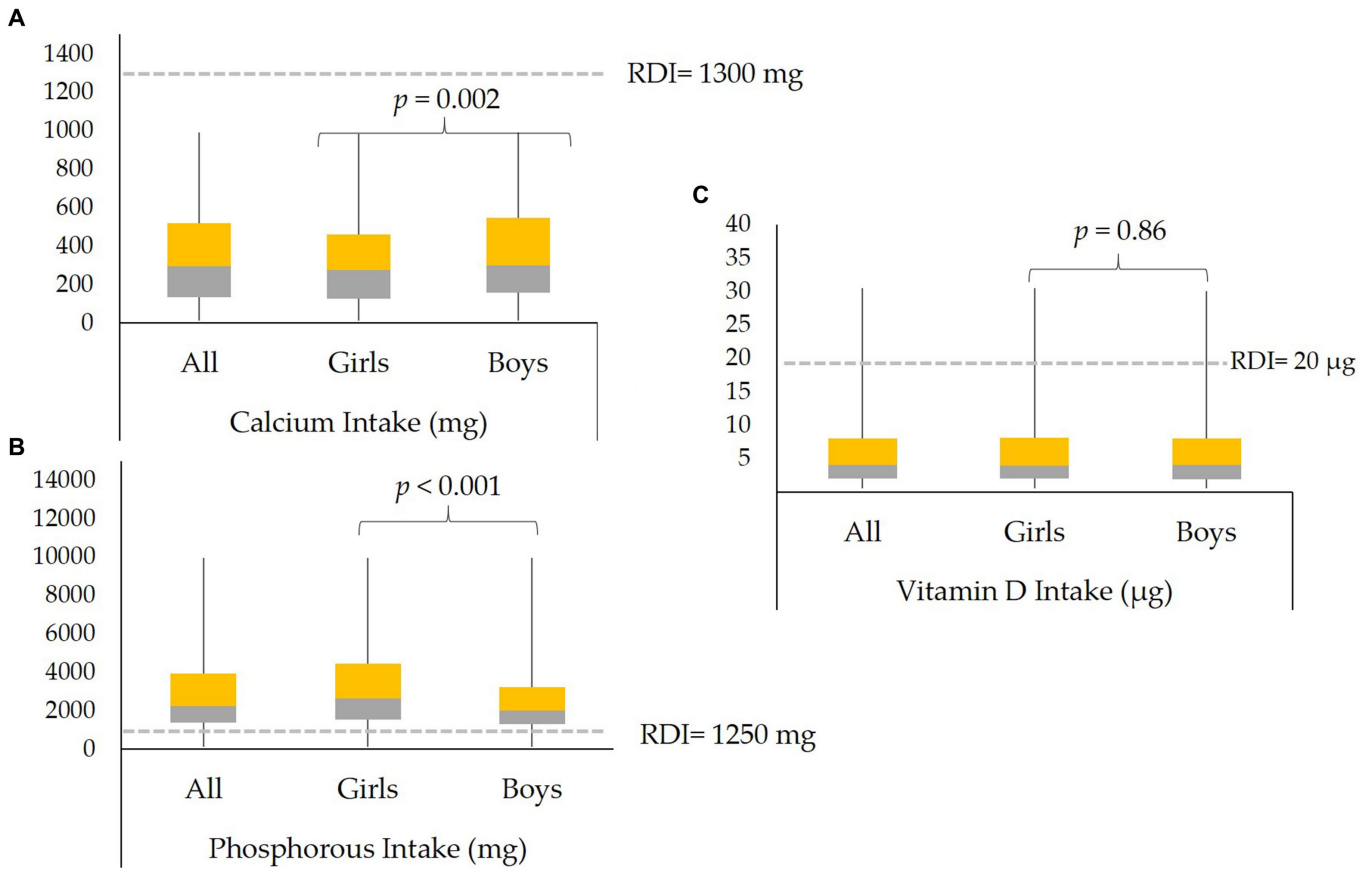
Median dietary intakes of **(A)** Ca, **(B)** Pi, and **(C)** Vitamin D in all participants and in boys and girls. The dashed gray lines indicate the RDI for each micronutrient.

**Figure 4:**
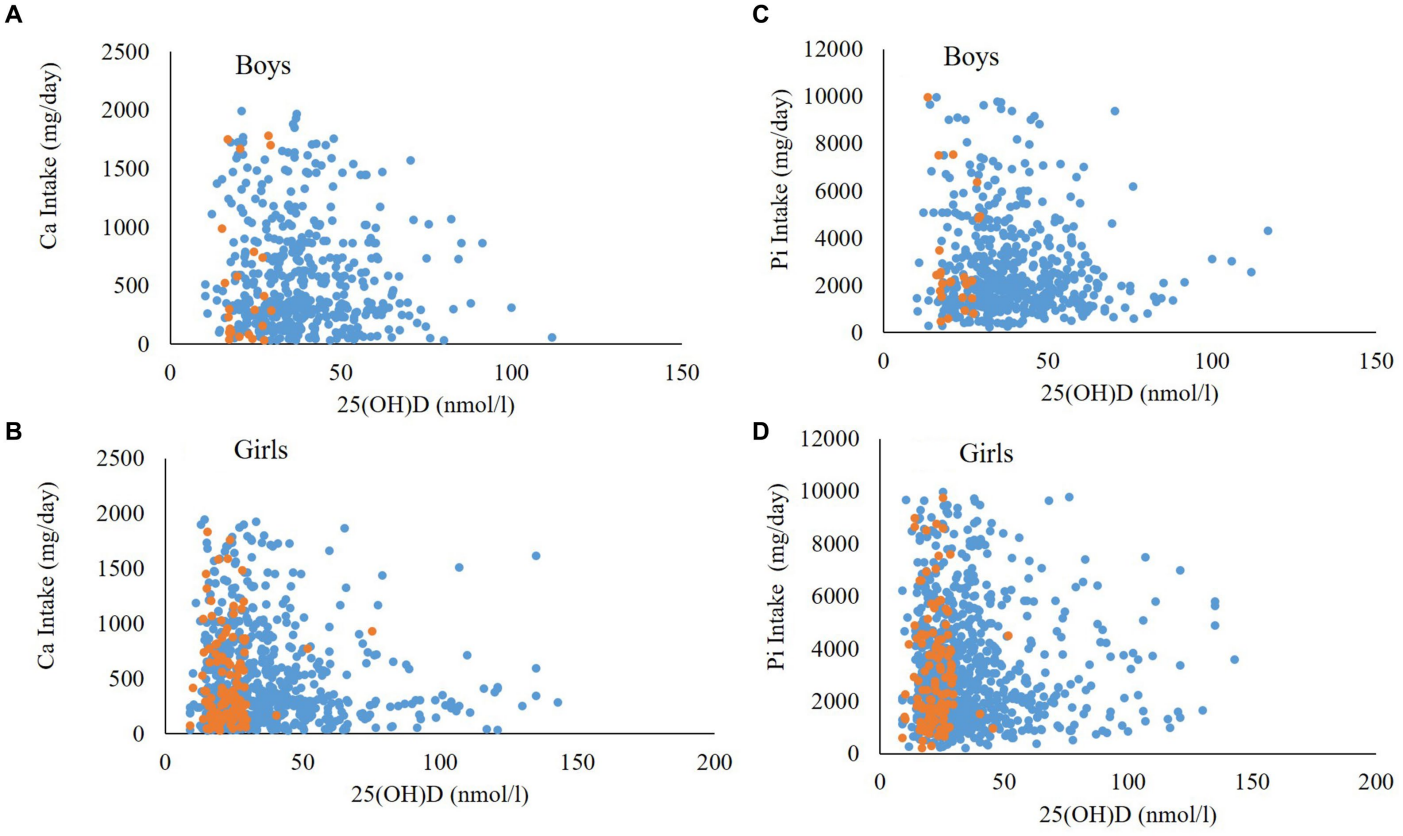
Associations between dietary Ca intake and 25(OH)D in **(A)** boys and **(B)** girls and Pi intake with 25(OH)D in **(C)** Boys and **(D)** Girls. Orange dots represent Biochemical OM cases while blue dots represent controls.

Figure 2A shows the box plot of ALP and 25(OH)D concentration according to biochemical OM status. Median concentrations of ALP (*p* < 0.001) and 25(OH)D (*p* < 0.001) were significantly lower in participants with biochemical OM as compared to controls. Figure 2B shows the mean serum concentrations of Ca (*p* < 0.001) and Pi (*p* < 0.001), both of which were significantly lower in participants with biochemical OM than controls. Figures 3A–C show the box plot of dietary Ca, Pi, and vitamin D intake among boys and girls, respectively. The median intake of Ca (*p* = 0.002) was significantly lower in boys than girls, while Pi intake (*p* < 0.001) was significantly lower in girls than boys. No significant difference in vitamin D intake was observed between boys and girls. Figure 4 shows the plotted associations between dietary Ca and Pi and boys and girls and showed no significant association with 25(OH)D.

Supplementary Table S2 shows the unadjusted anthropometric and clinical differences according to sex. Boys were significantly older (*p* < 0.001), have higher BMI (*p* < 0.001), waist (*p* < 0.001), WHR (*p* < 0.001), systolic BP (*p* < 0.001), Ca (*p* < 0.001), Pi (*p* < 0.001), ALP (*p* < 0.001), 25(OH)D (*p* < 0.001), and Fe (*p* < 0.001) as compared to girls. On the other hand, girls have higher hip circumference (*p* = 0.004) and diastolic BP (*p* < 0.001) as compared to boys.

Supplementary Table S3 shows the nutrients intake and % adequacy of participants according to sex. Results showed that boys reported significantly higher intake of dietary fat (*p* < 0.001), protein (*p* < 0.001), energy (*p* < 0.001), vitamin A (*p* < 0.001), thiamine (*p* = 0.018), riboflavin (*p* < 0.001), Na (*p* < 0.001), K (*p* < 0.001), Pi (*p* < 0.001), and Fe (*p* < 0.001) than girls. On the other hand, girls reported significantly higher intake of vitamin C (*p* = 0.05) and Ca (*p* < 0.001) as compared to boys. In both boys and girls, lower intake of vitamin D and Ca based on % adequacy were observed.

### 3.4. Significant dietary predictors of variations in markers of biochemical OM

Stepwise regression analysis was performed to identify which dietary nutrient were significantly contributing to the variations in circulating markers of biochemical OM (Supplementary Table S4). Results revealed that for all participants, thiamine was inversely correlated (*p* = 0.02) with serum Pi in all participants, explaining 0.2% of the variances in serum Pi (*r* = 0.05, adjusted *r*^2^ = 0.2%, *p* = 0.03). Furthermore, dietary K (*p* = 0.001), energy (*p* = 0.02), and vitamin A (*p* = 0.05) were inversely correlated with serum ALP while fiber (*p* < 0.001), thiamine (*p* = 0.001), and Ca (*p* = 0.001) were positively correlated with ALP, explaining 2.9% of the total variance (*r* = 0.18, adjusted *r*^2^ = 2.9%, *p* = 0.001). No significant predictors were found for serum Ca and 25(OH)D. Stratification according to biochemical OM status revealed no significant predictors for serum Pi, Ca and ALP. However, thiamine was inversely associated (*p* = 0.04) while protein was positively associated with (*p* = 0.002) serum 25(OH)D, with both variables explaining 4.3% of the variation in serum 25(OH)D (*r* = 0.23, adjusted *r*^2^ = 4.3%, *p* = 0.01) in the biochemical OM group. In controls, no significant predictors were found for serum Pi and Ca. However, CHO (*p* = 0.001), K (*p* < 0.001), and fat (*p* = 0.01) were inversely correlated with serum ALP while fiber (*p* < 0.001), thiamine (β *p* = 0.001), and Ca (*p* = 0.001) were positively correlated, cumulatively explaining 3.1% of the variation in serum ALP (*r* = 0.19, adjusted *r*^2^ = 3.1%, *p* < 0.001). Lastly, dietary vitamin C was positively associated (*p* = 0.02), while dietary vitamin D (*p* = 0.002) was positively correlated with serum 25(OH)D, explaining 0.6% of the total variation in serum 25(OH)D (*r* = 0.09, adjusted *r*^2^ = 0.6%, *p* = 0.004).

## 4. Discussion

The transition of dietary behavior from children to adolescents coincides with a period of more, but not full independence in decision making. It is a crucial period when food preferences are largely influenced by peers and advertisements on various platforms, and less by what is served at home (29). The present study aimed to determine the influence of dietary intakes on circulating biomarkers of hypomineralization among Arab adolescents and found a substantial nutrient imbalance in this population, most notably the very high consumption of foods rich in dietary Pi, Na, K, and Fe exceeding the RDI, with a concomitant very low intake of dietary Ca and vitamin D. We were able to demonstrate that the combination of low dietary Ca and low vitamin D status is associated with the biochemical OM signature. Furthermore, the significant associations of vitamin D status to most dietary intakes including dietary vitamin D were only apparent in the biochemical OM group, while the significant associations of serum ALP and Pi with dietary intakes elicited in all participants modestly varied but remained persistent even after stratification according to biochemical OM status.

The suboptimal nutrient intake of adolescents in the present study, that is, failing to meet the dietary recommendations, is similar to that reported in adolescent populations in industrialized or developing nations (30, 31). The abnormally high median levels of dietary Na, K, and Pi reflect the increased consumption of processed and frozen foods, junk foods and carbonated beverages, to name a few. These foods are known to contain these trace minerals in large amounts, particularly Pi derivatives which are abundant in fast foods (32). This is in sharp contrast with the very low intakes of dietary Ca and vitamin D independent of biochemical OM status. Dietary Ca in particular is significantly lower in boys than girls. Lower intakes of Ca and vitamin D have been more recently documented in a cohort of 631 Saudi adolescents aged 11–18 years (33). Excessive media use, which promotes sedentary lifestyle and negatively affects food choices is particularly common among Saudi adolescents (33), and aggravates this situation. Furthermore, the high consumption of phosphate-derivative foods such as processed foods and beverages confirm a previous survey among almost 11,000 Saudis especially in the young population (34). Interestingly, the low dietary Ca intake in Saudi adolescents extends to their adult counterparts already at risk of osteoporosis, with an average Ca intake of only 445 mg/day (RDI of 1,300 mg/day) (35).

It is worth noting that the influence of dietary intake on markers of bone mineralization appears to be more pronounced among adolescents with biochemical OM, suggesting a compensatory mechanism. Among participants with biochemical OM for instance, protein and thiamine intake were significant predictors of 25(OH)D variation. Fortunately, protein intake of participants was well above the RDI and well within limits for thiamine, suggesting that Arab adolescents consume large amounts of animal-based food in their diet which is compatible with the traditional Saudi diet. Due to the desert environment, vegetables and fruits are infrequently consumed within the region as a whole (36), and apart from dates, the population relies mainly on meat and carbohydrates as their main diet. Aggravated by the country’s current Westernisation, this unbalanced diet has progressively deteriorated to include high amounts of carbonated drinks (such as soda) and processed foods which, as mentioned, are high in Pi content (37). Despite the well-known inverse association between Ca and Pi in bone health (high Pi intake may decrease Ca absorption and high Ca may inhibit Pi uptake in the gut) (38), the elevated dietary Pi intake in our cohort did not protect from developing biochemical OM as the Ca-P product remained low in the OM group independent of Pi intake, indicating that association alone does not imply causation. The significant association of dietary vitamin D intake with serum 25(OH)D among only those without biochemical OM is worth noting since this association, while significant, was very small (*r*^2^ < 1%) in terms of affecting the participants’ vitamin D status, suggesting that other major factors (i.e., sun exposure, outdoor physical activity, vitamin D supplements) not assessed in the present study are largely influencing the vitamin D status of this population.

ALP was noted to be significantly and inversely associated with most macronutrients as well as dietary mineral intake in the present study, which can probably explain why in our previous study, elevated ALP was also the least common among the 4 markers of impaired mineralization (19). Although ALP isoenzymes were not measured in the present study, it has been observed in animal studies that high protein diet as well as high fat diet with vitamin D restriction can reduce ALP activity (39, 40). The elicited inverse associations of all dietary intake parameters with ALP observed in the present study should be interpreted with caution however, since ALP levels are expected to be high in adolescents, particularly boys, secondary to pubertal bone growth development (41).

Lastly, thiamine and protein were found to be predictors of 25(OH)D in the OM group in the present study, which, at 4% variance, is modest but still statistically significant. Since thiamine is not conventionally known to affect vitamin D metabolism, this association can be explained by the unhealthy dietary choices of participants. A similar study done among Brazilian adolescents aged 10–19 years found that processed and ultra-processed foods was associated with adequate intake of thiamine and inadequate intake of vitamin D and calcium, among others (42), demonstrating a comparable inverse association between thiamine and vitamin D. On the other hand, the significant association of protein intake to 25(OH)D cannot be fully explained and may require additional investigation detailing specific types of meats that held higher levels of vitamin D. Nevertheless, both protein and vitamin D intake are integral in musculoskeletal health, with the latter being a significant contributor in maintaining muscle mass and the former as an anabolic stimuli for muscle protein synthesis (5, 6).

The authors acknowledge several limitations. While the objectives were focused on differences and associations of dietary intake with biochemical OM and markers of impaired mineralization, other factors such as obesity, sunlight exposure and supplement intake were not considered, which could have a profound effect on these markers, 25(OH)D in particular since no observed differences were seen in the dietary vitamin D intake of both groups (43). Supplement intake in this age group however is low especially in females (44). Participants were recruited uniformly during summer time, which from previous observations translate to higher prevalence of vitamin D deficiency as compared to winter season in SA due to sun avoidance behaviour (45). Serum PTH was not assessed since prerequisites for analysis were not met. Hence, the association of PTH with dietary intake in this population was not captured. The operational diagnosis used for biochemical OM is only suggestive and not definitive, since histologic analysis for confirmation was not done. Lastly, the inherent memory recall bias in collecting dietary data, despite being performed by a dietitian, is a limitation. Nevertheless, the study is one of the largest of its kind to confirm whether the recent worsening dietary pattern in terms of quality, as observed among adolescents in the Middle East and SA in particular, is associated with biochemical OM. The findings emphasize the imperative need for food fortification with vitamin D and Ca to optimise nutrition in this vulnerable population who largely fail to meet the RDI (46–49). Additionally, the current national and regional guidelines for vitamin D supplementation are limited to the general adult population (50, 51). We recommend including the youngest and most vulnerable group of children and adolescents into these public health guidelines, in analogy to similar calls for political action worldwide (52, 53).

In conclusion, there is an alarming imbalance in the dietary intake of Saudi adolescents, with very low intakes of dietary Ca and vitamin D and high intakes of dietary Pi together with Na, K, and Fe. This imbalance modestly but significantly affects circulating markers of bone mineralization, contributing to the high prevalence of biochemical OM in Saudi adolescents, particularly girls. Food fortification of essential nutrients such as vitamin D and Ca may help reverse this nutrient disparity since supplementation alone has varying degrees of success.

## Data availability statement

The original contributions presented in the study are included in the article/Supplementary material, further inquiries can be directed to the corresponding authors.

## Ethics statement

The studies involving human participants were reviewed and approved by Institutional Review Board (IRB) approval was obtained from the College of Medicine, King Saud University, KSU, Riyadh, SA (E-21-6,095, approved 18 January 2019; amended 7 April 2022). Written informed consent to participate in this study was provided by the participants’ legal guardian/next of kin.

## Author contributions

NA, SU, and WH: conceptualization, critical revision, and data interpretation. SS: manuscript preparation. NA-D and SY: data collection. KW, SY, and NA-D: data analysis. SH: statistical analysis. All authors contributed to the article and approved the submitted version.

## Funding

This project was funded by the National Plan for Science, Technology, and Innovation (MAARIFAH), the King Abdulaziz City for Science and Technology, Kingdom of Saudi Arabia (award No. 2-17-03-001-0012).

## Conflict of interest

The authors declare that the research was conducted in the absence of any commercial or financial relationships that could be construed as a potential conflict of interest.

## Publisher’s note

All claims expressed in this article are solely those of the authors and do not necessarily represent those of their affiliated organizations, or those of the publisher, the editors and the reviewers. Any product that may be evaluated in this article, or claim that may be made by its manufacturer, is not guaranteed or endorsed by the publisher.

## Supplementary material

The Supplementary material for this article can be found online at: https://www.frontiersin.org/articles/10.3389/fnut.2023.1206711/full#supplementary-material
