# Supplementary material for: Association of bone mineralization markers with dietary nutrient intake in adolescents with and without biochemical osteomalacia
Source: Front Nutr. 2023 Jul 17;10:1206711. doi: 10.3389/fnut.2023.1206711 (PMC10388186; doi:10.3389/fnut.2023.1206711)
Supplement: Supplementary file 1 [file Data_Sheet_1.docx]

Supplementary Material

Associations of Mineralization Markers with Dietary Intakes with Markers in Adolescents with and without Biochemical Osteomalacia

**Nasser M. Al-Daghri^1*^, Shaun Sabico^1^, Kaiser Wani^1^, Syed Danish Hussain^1^, Sobhy Yakout^1^, Naji Aljohani^2,3^, Suma Uday^4,5#^, Wolfgang Högler^5,6*#^**

Joint Correspondence: Nasser M. Al-Daghri, ndaghri@ksu.edu.sa; Wolfgang Högler, [wolfgang.hoegler@kepleruniklinikum.at](mailto:wolfgang.hoegler@kepleruniklinikum.at)

# #joint senior authors

# Supplementary Tables

**Supplementary table 1**. Age- and sex-specific normal ranges used for serum alkaline phosphatase (ALP) and inorganic phosphorous (Pi)

| **Age groups (years)** | **Boys** | **Girls** |
| --- | --- | --- |
| ALP (U/l) (27) * |  |  |
| 1-9 | 156-369 | 156-369 |
| 10-12 | 141-460 | 141-460 |
| 13-14 | 127-517 | 62-280 |
| 15-16 | 89-365 | 54-128 |
| 17-18 | 59-164 | 48-95 |
| Pi (mmol/l) (28) |  |  |
| 5–13 | 1.19–1.74 | 1.29–1.68 |
| 14–15 | 1.13–1.52 | 1.13–1.58 |
| 16–17 | 1.0–1.52 | 1.0–1.52 |

*References ranges established from Abbott ARCHITECT analyser.

**Supplementary table 2**: Anthropometric and biochemical characteristics of study participants

| **Parameters** | **Girls (N=1083)** | **Boys (N=736)** | **P-value** |
| --- | --- | --- | --- |
| Age (years) | 14.8 ± 1.8 | 15.3 ± 1.6 | <0.001 |
| Height (cm) | 157.0 ± 9.6 | 163.2 ± 10.0 | <0.001 |
| Weight (kg) | 58.4 ± 15.0 | 65.7 ± 17.5 | <0.001 |
| BMI (kg/m^2^) | 23.6 ± 5.8 | 24.5 ± 5.6 | <0.001 |
| BMI Z-score | 0.0 ± 0.8 | 0.0 ± 1.2 | 0.77 |
| Waist (cm) | 72.0 ± 11.0 | 75.0 ± 21.0 | <0.001 |
| Hips (cm) | 89.0 ± 14.0 | 83.0 ± 22.0 | 0.004 |
| WHR | 0.8 ± 0.1 | 0.9 ± 0.1 | <0.001 |
| Systolic BP (mmHg) | 114.0 ± 18.0 | 119.0 ± 13.0 | <0.001 |
| Diastolic BP (mmHg) | 74.0 ± 12.0 | 69.0 ± 11.0 | <0.001 |
| Ca (mmol/l) | 2.5 ± 0.3 | 2.6 ± 0.3 | <0.001 |
| Pi (mmol/l) | 1.4 ± 0.3 | 1.5 ± 0.5 | <0.001 |
| ALP (U/l) | 58.3 (40.4 - 86.2) | 73.1 (49.8 - 101.5) | <0.001 |
| 25(OH) D (nmol/l) | 27.8 (20.7 - 40.2) | 35.5 (27.8 - 45.9) | <0.001 |
| ALT (U/l) | 10.5 (8.0 - 15.1) | 12.4 (8.4 - 16.6) | 0.02 |
| Fe (µg/l) | 753.8 (510.4 - 1038.7) | 944.9 (712.5 - 1185.9) | <0.001 |

Note: Data presented as mean ± standard deviation and median (Q1- Q3) for normal and non-normal continuous variables. The difference between the sexes is significant for p<0.05.

**Supplementary Table 3**. Nutrient intake according to sex

| **Parameters** | **RDI*** | **Girls (N=1083)** | | | **Boys (N=736)** | | | **P-value** |
| --- | --- | --- | --- | --- | --- | --- | --- | --- |
|  |  | **Intake** | | **Adequacy (%)** | **Intake** | | **Adequacy (%)** |  |
|  |  | **Median (IQR)** | **Mean (SE)** | **Mean (SE)** | **Median (IQR)** | **Mean (SE)** | **Mean (SE)** |  |
| Energy (Kcal) | 2000 | 2899.2 (1719-4758) | 8004.6 (778.3) | 400.2 (38.9) | 3513.1 (1810-6441) | 6287.0 (691.6) | 314.4 (34.6) | <0.001 |
| Fat (g) | 78g | 126.3 (70-256) | 488.1 (57.4) | 625.8 (73.5) | 182.7 (94-344) | 363.5 (48.1) | 466.6 (61.7) | <0.001 |
| Protein (g) | 50g | 103.8 (66-170) | 369.2 (62.6) | 739.8 (125.4) | 129.4 (68-240) | 312.0 (51.8) | 624.0 (103.5) | <0.001 |
| CHO (g) | 275g | 419.7 (217-676) | 703.2 (32.9) | 255.7 (12.0) | 436.1 (215-796) | 587.5 (29.5) | 213.6 (10.7) | 0.06 |
| Fiber (g) | 28g | 30 (14-51) | 46.5 (1.9) | 169.9 (7.0) | 28.8 (15-56) | 42.7 (2.7) | 152.9 (9.7) | 0.42 |
| **Minerals** | |  |  |  |  |  |  |  |
| Na (mg) | 1500mg | 3428.5 (2051-6315) | 9086.7 (524.4) | 605.8 (35.0) | 4891.8 (2667-10089) | 6758.4 (560.7) | 450.6 (37.4) | <0.001 |
| K (mg) | 4700mg | 5510.4 (3008-9304) | 12603.2 (934.5) | 268.2 (19.9) | 6991.3 (3373-12655) | 9653.5 (779.3) | 205.4 (16.6) | <0.001 |
| Ca (mg/day) | 1300mg | 299.9 (158-546) | 577.1 (48.7) | 44.7 (3.8) | 275.6 (127-462) | 663.6 (43.4) | 51.4 (3.4) | 0.002 |
| Pi (mg) | 1250mg | 1997.4 (1325-3236) | 5535.6 (632.5) | 442.8 (50.6) | 2657.5 (1538-4441) | 4501.3 (533.0) | 360.1 (42.6) | <0.001 |
| Fe (mg) | 18mg | 34.1 (18-63) | 86.6 (9.4) | 492.7 (53.6) | 49 (21-84) | 70.1 (8.8) | 399.5 (50.2) | <0.001 |
| **Vitamins** |  |  |  |  |  |  |  |  |
| Vitamin A (µg) | 900µg | 765 (432-1706) | 2276.9 (101.4) | 253.0 (11.3) | 1277 (559-2615) | 1683.5 (114.6) | 187.1 (12.7) | <0.001 |
| Thiamine (mg) | 1.2mg | 1.5 (0.9-3) | 4.3 (0.6) | 401.2 (57.9) | 1.8 (0.9-3.6) | 5.3 (0.6) | 497.1 (55.7) | 0.02 |
| Riboflavin (mg) | 1.3mg | 3.2 (2-7.4) | 14.9 (3.6) | 1174.3 (284.9) | 5.0 (2.6-10) | 12.5 (3.1) | 985.6 (242.6) | <0.001 |
| Vitamin B12 (µg) | 2.4µg | 10.4 (5-20) | 76.7 (39.5) | 3320.7 (1710.0) | 10.1 (4.5-22) | 82.4 (33.0) | 3482.2 (1396.1) | 0.24 |
| Vitamin C (mg) | 90mg | 128.0 (46-259) | 201.3 (12.9) | 226.2 (14.5) | 102.6 (42-225) | 214.4 (16.4) | 240.0 (18.4) | 0.05 |
| Vitamin D (µg) | 20µg | 4.1 (2-8) | 8.2 (0.9) | 45.5 (5.2) | 3.9 (2-8) | 7.9 (0.8) | 42.0 (4.1) | 0.86 |

**Note**: Data presented as Median (Q1 – Q3); P-value obtained from Mann-Whitney U test; p<0.05 considered significant; *Based on reference caloric intake (2,000 calories) for adults and children aged 4 years and above (#)

**Supplementary Table 4. Significant Predictors**

|  | **Ca** | **Pi** | **ALP** | **25(OH)D** |
| --- | --- | --- | --- | --- |
| **All Participants** |  |  |  |  |
| Significant Predictors | None | Thiamine (-0.05) | K (-0.14)  Thiamine (0.14)  Ca (0.10)  Energy (-0.12)  Fiber (0.16)  Vitamin A (-0.09) | None |
| Adjusted R^2^ | - | 0.2% | 2.9% | - |
| *p*-value | - | 0.03 | <0.001 | - |
| **Biochemical OM** |  |  |  |  |
| Significant Predictors | None | None | None | Protein (0.35)  Thiamine (-0.24) |
| Adjusted R^2^ | - | - | - | 4.3% |
| *p*-value | - | - | - | 0.01 |
| **Control** |  |  |  |  |
| Significant Predictors | None | None | Ca (0.11)  Pi (-0.24)  Fiber (0.21)  Thiamine (0.14)  CHO (-0.16) | Vitamin D (0.10)  Vitamin C (0.07) |
| Adjusted R^2^ | - | - | 3.1% | 0.6% |
| *p-*value | - | - | <0.001 | 0.004 |

**Note**: Significant predictors were presented as parameter (Standardized β coefficients as obtained from stepwise regression analysis); *p*< 0.05 considered as significant.
